# *GhMAP3K65*, a Cotton Raf-Like MAP3K Gene, Enhances Susceptibility to Pathogen Infection and Heat Stress by Negatively Modulating Growth and Development in Transgenic *Nicotiana benthamiana*

**DOI:** 10.3390/ijms18112462

**Published:** 2017-11-21

**Authors:** Na Zhai, Haihong Jia, Dongdong Liu, Shuchang Liu, Manli Ma, Xingqi Guo, Han Li

**Affiliations:** State Key Laboratory of Crop Biology, College of Life Sciences, Shandong Agricultural University, Tai’an 271018, China; zhaina@sdau.edu.cn (N.Z.); jiahh@sdau.edu.cn (H.J.); darcy.liu@olamnet.com (D.L.); liushuchang1992@gmail.com (S.L.); ManliMa@sdau.edu.cn (M.M.); xqguo@sdau.edu.cn (X.G.)

**Keywords:** cotton, VIGS, ectopic expression, pathogen infection, thermotolerance

## Abstract

Mitogen-activated protein kinase kinase kinases (MAP3Ks), the top components of MAPK cascades, modulate many biological processes, such as growth, development and various environmental stresses. Nevertheless, the roles of MAP3Ks remain poorly understood in cotton. In this study, *GhMAP3K65* was identified in cotton, and its transcription was inducible by pathogen infection, heat stress, and multiple signalling molecules. Silencing of *GhMAP3K65* enhanced resistance to pathogen infection and heat stress in cotton. In contrast, overexpression of *GhMAP3K65* enhanced susceptibility to pathogen infection and heat stress in transgenic *Nicotiana benthamiana*. The expression of defence-associated genes was activated in transgenic *N. benthamiana* plants after pathogen infection and heat stress, indicating that *GhMAP3K65* positively regulates plant defence responses. Nevertheless, transgenic *N. benthamiana* plants impaired lignin biosynthesis and stomatal immunity in their leaves and repressed vitality of their root systems. In addition, the expression of lignin biosynthesis genes and lignin content were inhibited after pathogen infection and heat stress. Collectively, these results demonstrate that *GhMAP3K65* enhances susceptibility to pathogen infection and heat stress by negatively modulating growth and development in transgenic *N. benthamiana* plants.

## 1. Introduction

Unlike animals, plants are sessile and cannot change their location to flee unfavourable environmental conditions. Nevertheless, they have evolved a series of sophisticated mechanisms to perceive biotic and abiotic stresses, and to translate this perception into a timely and effectively adaptive response [1,2]. Mitogen-activated protein kinase (MAPK) cascades are highly conserved signal transduction pathways found in all eukaryotes and are often involved in plant response mechanisms [3]. Pathways involving MAPK cascades can trigger different cellular activities, including responses to environmental stresses and programmed cell death and so on [4,5].

MAPK cascades are composed of three functional protein kinases: MAPK kinase kinases, MAPK kinases and MAPKs [3]. These kinases are sequentially activated through phosphorylation by their upstream components [6]. Based on the sequence of their kinase catalytic domain, MAP3Ks are classified into three groups: the MEKK-like, ZIK-like and Raf-like families [7]. While Raf-like MAP3Ks constitute largest MAP3K subfamily, only a small number of Raf-like MAP3Ks have been studied [2].

An increasing number of studies suggest that Raf-like MAP3Ks play significant roles in plant growth and development as well as in the responses of specific plant hormone signal transduction pathways to various environmental stresses [8,9]. The biological importance of Raf-like MAP3Ks has been highlighted by functional analyses. Raf-like MAP3K genes have been reported to participate in multiple biological processes. For example, *MAP3Kδ4* is crucial for regulating plant growth and shoot branching [10], and *MAP3Kδ4*-overexpression increases salt stress tolerance and plays a significant role in the ABA signalling pathway in *Arabidopsis* [11]. *GhMAP3K40* mediates the reduced tolerance to biotic and abiotic stresses in *N. benthamiana* by negatively regulating growth and development [12]. A growing number of studies demonstrate that Raf-like MAP3Ks are involved in the responses to various environmental stresses and in modulating plant growth and development.

Cotton is suitable for growing in warm seasons, and its growth and yield are seriously threatened by various environmental stresses. Diseases generally cause significant losses in cotton production, particularly at high temperature and high humidity [13]. Disease resistance regulated by *SLH1* and *RPW8* during plant growth and development is inhibited at high temperature and high humidity [14,15]. Nevertheless, whether this phenomenon affects additional genes remains unclear. Crosstalk mechanisms are involved in the responses to disease, high temperature and high humidity conditions [13]. Three plant hormones, salicylic acid (SA), jasmonate (JA), and ethylene (ET), are known to play significant roles in regulating plant defence responses to various pathogens and abiotic stresses, such as heat [16,17,18,19].

Previous studies have largely focused on MAP2Ks and MAPKs in cotton, while findings regarding MAP3Ks, particularly Raf-like MAP3Ks, are limited. In this study, the expression profiles of *GhMAP3K65* under different environmental stresses or treatment with multiple molecules were examined in cotton. Silencing of *GhMAP3K65* enhanced resistance to pathogen infection and heat stress in cotton. In contrast, ectopic expression of *GhMAP3K65* in *N. benthamiana* enhanced susceptibility to pathogen infection and heat stress by negatively modulating plant growth and development. Nevertheless, because overexpression of *GhMAP3K65* activated the transcription of defence-associated genes, *GhMAP3K65* played a positive role in the responses to pathogen infection and heat stress. These findings broaden our horizon that Raf-like MAP3Ks may play significant roles in signal transduction.

## 2. Results

### 2.1. Isolation and Sequence Analysis of GhMAP3K65

Based on the conserved Raf-like MAP3K region, the internal *GhMAP3K65* fragment was obtained. The 5′-untranslated region (UTR) and 3′-UTR were identified via rapid amplification of cDNA ends polymerase chain reaction (RACE–PCR). Full-length *GhMAP3K65* cDNA consisted of 1374 nucleotides, including a 1143 bp open reading frame (ORF), a 68 bp 5′-UTR and a 163 bp 3′-UTR. *GhMAP3K65* ORF encoded a protein of 381 amino acid residues, with a predicted molecular mass and isoelectric point of 45.64 and 7.13 kDa, respectively.

As shown in Figure S1a, multiple sequence alignment demonstrated that the amino acid sequence of GhMAP3K65 was similar to that of other typical plant Raf-like MAP3Ks, showing high homology to AtRaf39 from *Arabidopsis thaliana* (73.30%), BnaRaf39 from *Brassica napus* L. (71.73%), OsMAP3K27 from *Oryza sativa* L. (71.20%), and ZmMAP3K50 from *Zea mays* L. (69.82%). Moreover, GhMAP3K65 possessed typical characteristics of MAPK, including a catalytic protein kinase domain between residues 104–386. The catalytic protein kinase domain of GhMAP3K65 included an ATP-binding site (IAHGTYGTVY), a Ser/Thr kinase active site (IVHRDVKTENMLL), and a conserved sequence (GTLGYMAPEVL). GhMAP3K65 was highly similar to the Raf-like MAP3Ks AtRaf39, BnaRaf39, OsMAP3K27, and ZmMAP3K50 (Figure S1b). These results indicate that GhMAP3K65 is a Raf-like MAP3K gene.

### 2.2. GhMAP3K65 is Localized to the Cytoplasm and Nucleus

**Figure 1:**
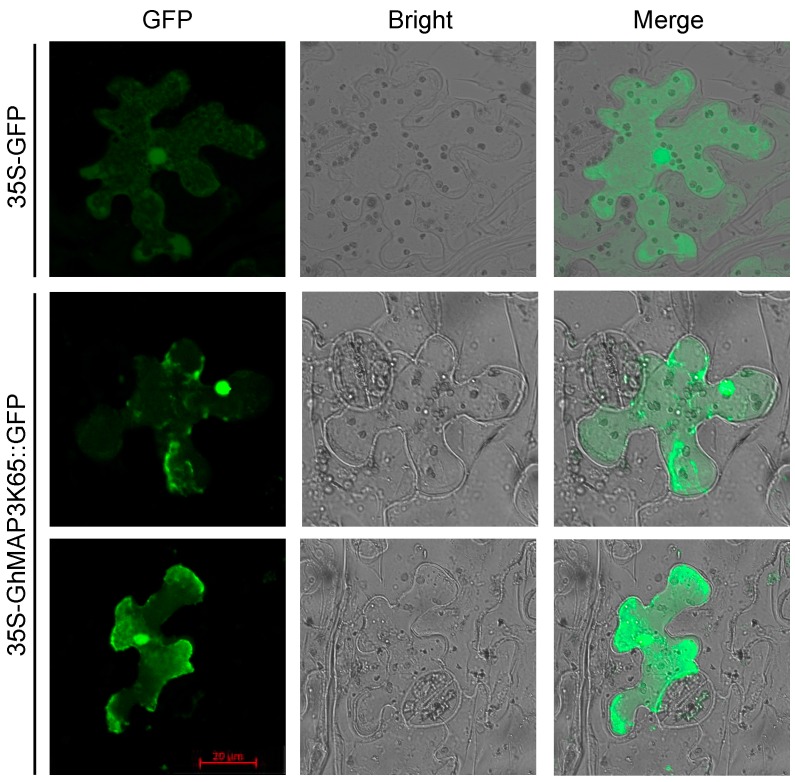
Subcellular localization of GhMAP3K65 protein transiently expressed in *N. benthamiana* cells. The 35S-GFP and 35S-GhMAP3K65::GFP were transiently expressed in epidermal cells and the resulting green fluorescence was detected using two-photon laser confocal microscope.

To confirm subcellular localization of GhMAP3K65, GhMAP3K65 ORF without stop codon was fused to 5′-terminal end of green fluorescent protein (GFP) to construct pROKII-GhMAP3K65-GFP, which was regulated by the cauliflower mosaic virus 35S (CaMV35S) promoter. Next, 35S-GFP and 35S-GhMAP3K65::GFP were transformed into *N. benthamiana* cells, and fluorescence was observed with two-photon laser confocal microscope. As shown in Figure 1, GFP control was localized to the cytoplasm and nucleus and GhMAP3K65::GFP fusion protein was detected throughout cytoplasm and nucleus in *N. benthamiana* epidermal cells. These results suggest that GhMAP3K65 is both a cytoplasmic- and nuclear-localized protein.

The localization patterns for the 35S-GFP and 35S-GhMAP3K65::GFP proteins were the same. To determine the integrity of 35S-GhMAP3K65::GFP protein, Western blot analyses of the 35S-GFP and 35S-GhMAP3K65::GFP proteins from transiently transformed *N. benthamiana* plants were examined using a GFP antibody (Figure S2), revealing that the 35S-GhMAP3K65::GFP protein is integral.

### 2.3. Relative Expression Profiles of GhMAP3K65

**Figure 2:**
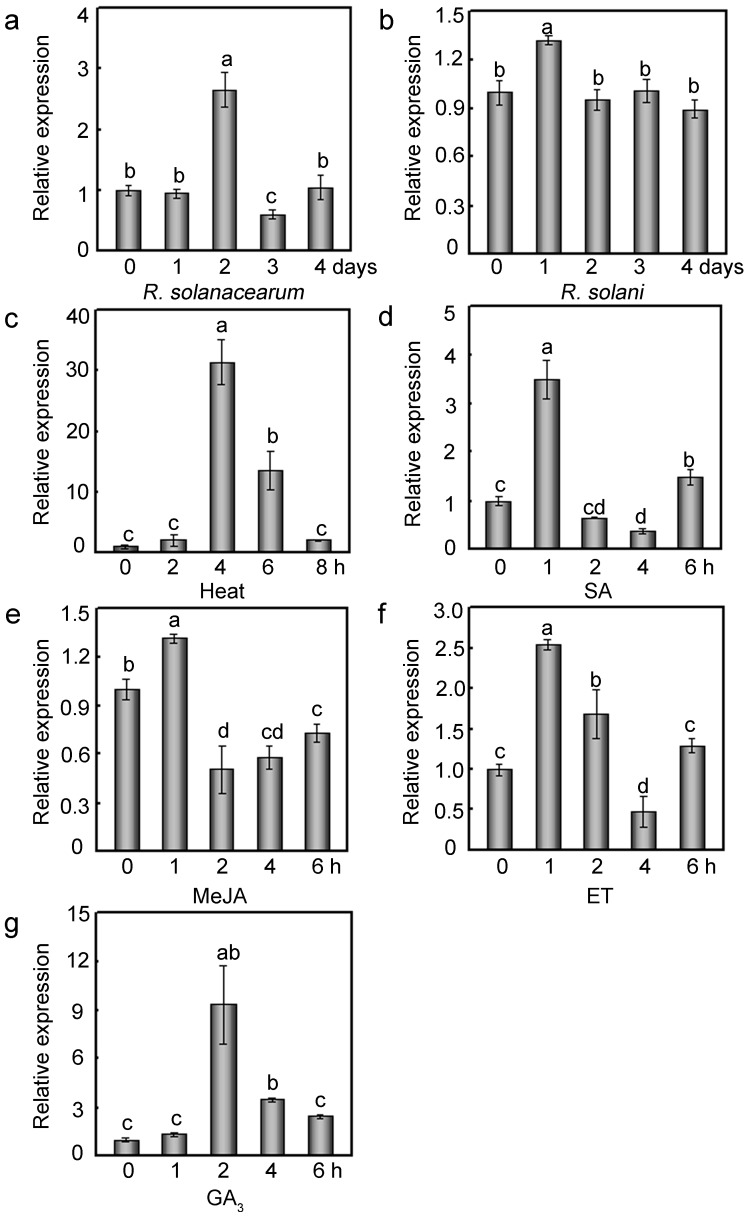
Expression profiles of *GhMAP3K65* in cotton. Seven-day-old cotton seedlings were examined by the following treatments: (**a**,**b**) Seedlings were inoculated with *R. solanacearum* and *R. solani* at 8 o’clock a.m. using root-dipping method, and cotton cotyledons were collected every day at 8 o’clock a.m.; (**c**) Seedlings were exposed to heat stress in a 42 °C incubator at 100% humidity and darkness at 8 o’clock a.m.; (**d**–**g**) Cotton cotyledons were sprayed with 2 mM SA, 100 µM MeJA, 5 mM ET, and 500 µM GA_3_ at 8 o’clock a.m. Regarding the sample collection time points, 8 o’clock a.m. correspond to 0 h, 9 o’clock a.m. correspond to 1 h, etc. The expression profiles of *GhMAP3K65* were determined via quantitative real-time PCR (qRT-PCR). Total RNA was extracted from cotton cotyledons at the indicated time points. *GhUBI* (GenBank accession number: EU304080) was used as an internal control, and the experiments were repeated at least three times. Different letters above the columns indicate significant differences (*p* < 0.05) according to Duncan’s multiple range test.

To determine the putative role of *GhMAP3K65* in the responses to various environmental stresses, its expression profile was studied after exposure to biotic and abiotic stresses. The *GhMAP3K65* expression profile in cotton plants not exposed to treatment was used as a mock control, and no changes in *GhMAP3K65* transcript levels were observed during the 0–8 h series (Figure S3). *GhMAP3K65* expression was notably increased by *Ralstonia solanacearum* and mildly induced by *Rhizoctonia solani* (Figure 2a,b). Heat treatment (42 °C) markedly enhanced *GhMAP3K65* expression (Figure 2c). In addition, to explore the mechanism underlying *GhMAP3K65* signal transduction in the responses to biotic and abiotic stresses, the effects of diverse signalling molecules on *GhMAP3K65* expression were examined. *GhMAP3K65* expression was enhanced by SA, methyl jasmonate (MeJA), and ET (Figure 2d–f) and was dramatically induced by gibberellic acid (GA_3_) (Figure 2g). These results suggest that *GhMAP3K65* may be involved in response to biotic and abiotic stresses by mediating plant defence signal transduction pathways.

### 2.4. Effects of GhMAP3K65 Silencing on Pathogen Infection and Heat Stress in Cotton

**Figure 3:**
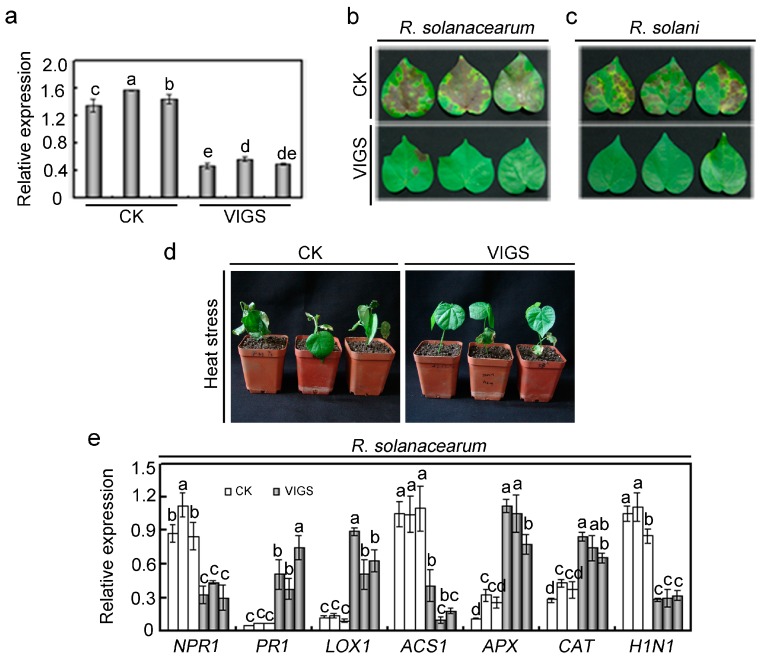

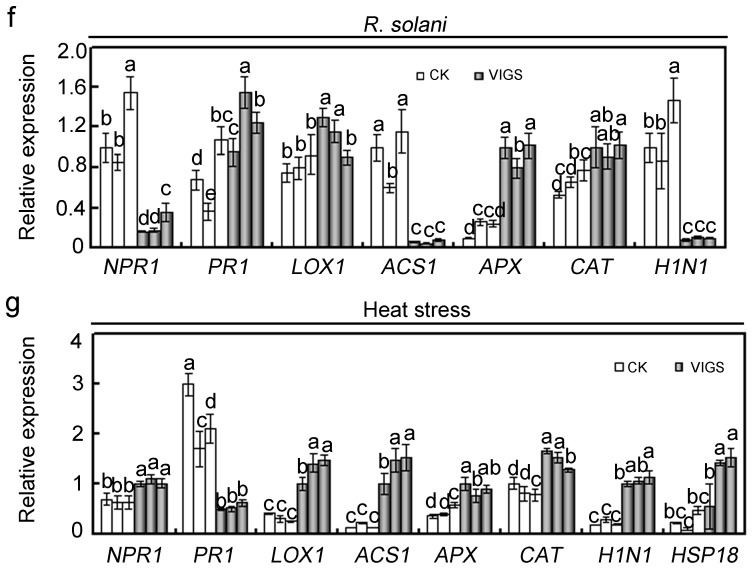
Loss-of-function analysis of *GhMAP3K65* in cotton. (**a**) Relative *GhMAP3K65* transcript levels in vector-treated (CK) and *GhMAP3K65*-silenced (VIGS) cotton plants were examined via qRT-PCR; (**b**–**d**) Representative phenotypes of CK and VIGS plants after *R. solanacearum*, *R. solani* and heat stress, respectively; (**e**–**g**) The transcript levels of some cotton pathogenesis markers were examined by qRT-PCR 7 days after pathogen infection and 24 h after heat stress, respectively. Different letters above the columns indicate significant differences (*p* < 0.05) according to Duncan’s multiple range test.

Differential expression profile analysis demonstrated that *GhMAP3K65* may play roles in multiple plant defence responses. To evaluate the function of *GhMAP3K65* in response to pathogen infection and heat stress, the virus-induced gene silencing (VIGS) technique was used to knock down *GhMAP3K65* expression in cotton [20]. The transcript levels of *GhMAP3K65* were examined via qRT-PCR in empty vector-treated (CK) and *GhMAP3K65*-silenced (VIGS) cotton plants. The decreased expression of *GhMAP3K65* demonstrated that *GhMAP3K65* had been successfully knocked down in cotton (Figure 3a). To confirm pathogen infection and heat stress phenotypes of *GhMAP3K65*-silenced cotton, CK and VIGS plants were tested. After pathogen infection, VIGS leaves showed enhanced resistance compared with CK (Figure 3b,c). Similarly, CK plants exhibited reduced thermotolerance after heat stress (Figure 3d). We obtained similar results with two additional CK and VIGS plants (Figure S4). These results suggested that silencing of *GhMAP3K65* clearly enhanced resistance to pathogen infection and heat stress.

Furthermore, to explore mechanisms underlying effects of *GhMAP3K65* silencing on pathogen infection and heat stress, the expression patterns of SA/JA/ET-mediated genes, reactive oxygen species (ROS) detoxification-related genes, hypersensitive response (HR)-related genes, and heat shock-related genes in CK and VIGS plants after pathogen infection and heat stress were examined by qRT-PCR. After pathogen infection, up-regulated genes included *PR1* (pathogenesis-related protein 1), *APX* (ascorbate peroxidase ), *CAT* (catalase), and *LOX1* (lipoxygenase 1), whereas the down-regulated transcription of *NPR1* (natriuretic peptide receptor 1), *ACS1* (1-aminocyclopropane-1-carboxylate synthase 1), and *H1N1* (hairpin-induced 1) was observed (Figure 3e,f). As shown in Figure 3g, the transcript levels of *NPR1*, *LOX1*, *ACS1*, *APX*, *CAT*, *H1N1,* and *HSP18* (heat shock protein 18) were significantly increased in VIGS plants compared with those in CK plants, nevertheless the transcript level of *PR1* in VIGS plants was obviously decreased relative to wild type (WT) plants after heat stress. These results indicated that *GhMAP3K65* may respond to pathogen infection and heat stress by SA/JA/ET and ROS signalling pathways.

### 2.5. Overexpression of GhMAP3K65 Enhanced Susceptibility to Pathogen Infection and Heat Stress

**Figure 4:**
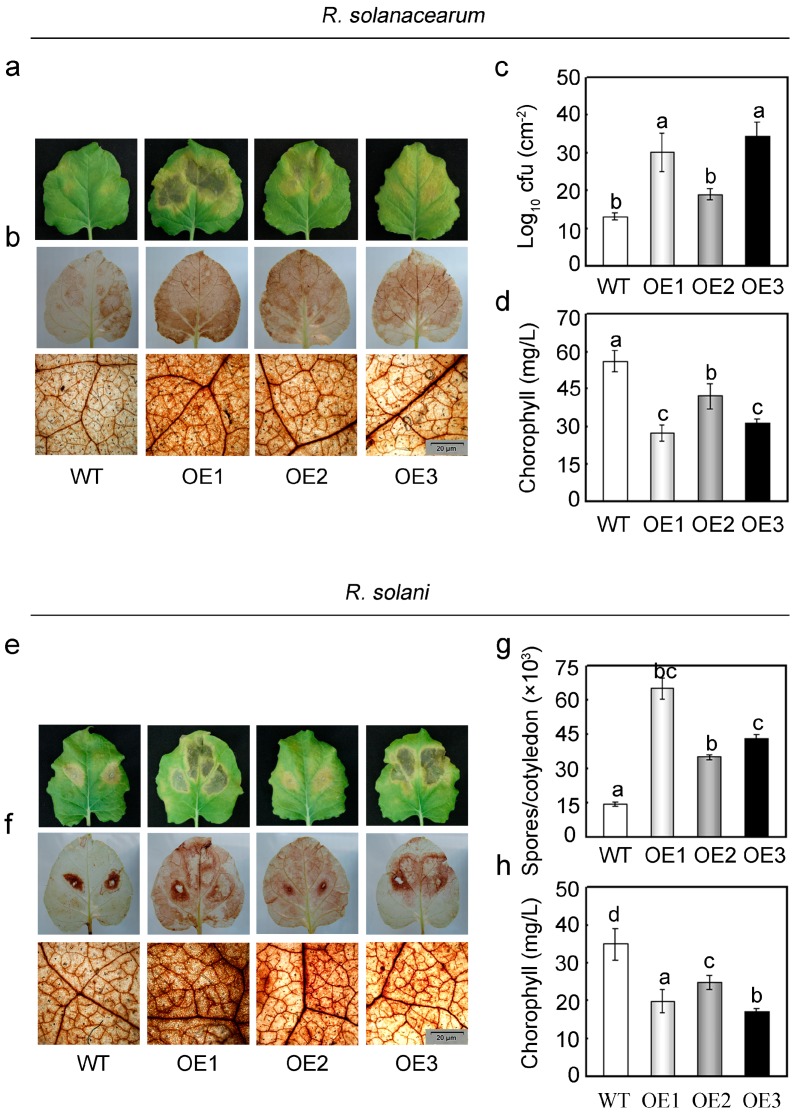
Pathogen infection of *GhMAP3K65*-overexpressing plants. (**a**,**e**) Symptoms in detached leaves of WT and *GhMAP3K65*-overexpressing plants in response to *R. solanacearum* and *R. solani* infection, respectively. The detached leaves were photographed 7 days after infection; (**b**,**f**) Symptoms in detached leaves of WT and *GhMAP3K65*-overexpressing plants following *R. solanacearum* and *R. solani* infection, respectively. The accumulation of H_2_O_2_ was detected via 3,3′-diaminobenzidine (DAB) staining; (**c**) Bacterial growth in the leaves of WT and *GhMAP3K65*-overexpressing plants after *R. solanacearum* infection; (**g**) The number of spores per cotyledon after *R. solani* infection; (**d**,**h**) Chlorophyll contents were measured after *R. solanacearum* and *R. solani* infection, respectively. The experiments were repeated at least three times. Different letters above the columns indicate significant differences (*p* < 0.05) according to Duncan’s multiple range test.

To confirm the results of *GhMAP3K65* silencing observed in cotton in a corresponding experimental system, three transgenic *N. benthamiana* lines (OE1–OE3) were randomly selected for subsequent functional analyses, as transforming cotton is difficult and time-consuming. To explore whether *GhMAP3K65*-overexpression influences resistance to pathogen infection and heat stress in *N. benthamiana*, phenotypes of transgenic *N. benthamiana* plants were examined. For pathogen infection, detached leaves of WT and *GhMAP3K65*-overexpressing plants were injected with *R. solanacearum* and *R. solani*, and those of *GhMAP3K65*-overexpressing plants showed more severe chlorosis and larger lesions after 7 days (Figure 4a,e). Pathogen infection often results in production of ROS, which play important roles in defence response [21,22]. In diverse ROS, H_2_O_2_ is the only species that can cross plant membranes and thus directly functions in cell-to-cell signalling. To examine whether ROS production was relevant to enhanced susceptibility of transgenic *N. benthamiana* plants, H_2_O_2_ accumulation was monitored using 3,3′-diaminobenzidine (DAB) staining. After pathogen infection, H_2_O_2_ contents of the overexpressing plants were higher than those of WT plants (Figure 4b,f). These results indicated that *GhMAP3K65*-overexpression could enhance defence susceptibility by inducing the production of pathogen-induced ROS (mainly H_2_O_2_).

To precisely quantify degree of pathogen infection in leaves, colony-forming units (cfu), spores per cotyledon and chlorophyll content assays were performed after pathogen infection. Compared with WT plants, the transgenic *N. benthamiana* plants exhibited increased bacterial growth, more spores (Figure 4c,g) and lower chlorophyll contents (Figure 4d,h). These results indicated that *GhMAP3K65*-overexpressing plants suffered more serious damage after pathogen infection.

**Figure 5:**
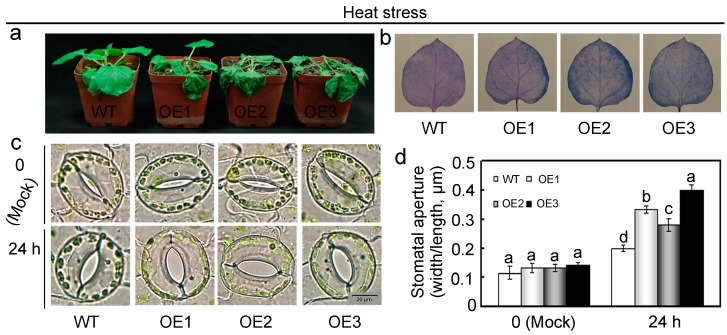
Heat stress response of *GhMAP3K65*-overexpressing plants. (**a**) Photographs of WT and *GhMAP3K65*-overexpressing plants grown in soil under heat stress for 24 h; (**b**) The accumulation of dead cells was detected via Trypan blue staining; (**c**,**d**) Stomatal changes under heat stress were observed using a microscope. The experiments were repeated at least three times. The different letters above the columns indicate significant differences (*p* < 0.05) according to Duncan’s multiple range test.

To evaluate the impact of *GhMAP3K65* on thermotolerance, WT and transgenic *N. benthamiana* plants were exposed to heat stress in a 42 °C incubator for 24 h at 100% humidity and darkness. *GhMAP3K65*-overexpressing plants displayed more severe heat-induced cell death than WT plants (Figure 5a). In addition, local defence response was examined via Trypan blue staining to detect HR-like cell death after heat stress. Compared with WT plants, the transgenic *N. benthamiana* plants showed significantly increased blue area (HR-like cell death) under heat stress (Figure 5b). Degree of stomatal opening in *GhMAP3K65*-overexpressing plants was not significantly different from that in WT plants under normal conditions. Nevertheless, extent of stomatal opening observed in *GhMAP3K65*-overexpressing plants was greater than that in WT plants after heat stress treatment (Figure 5c,d). These results indicated that *GhMAP3K65*-overexpressing plants suffered greater damage after heat stress.

### 2.6. Overexpression of GhMAP3K65 Activated Transcription of Defence-Related Genes

**Figure 6:**
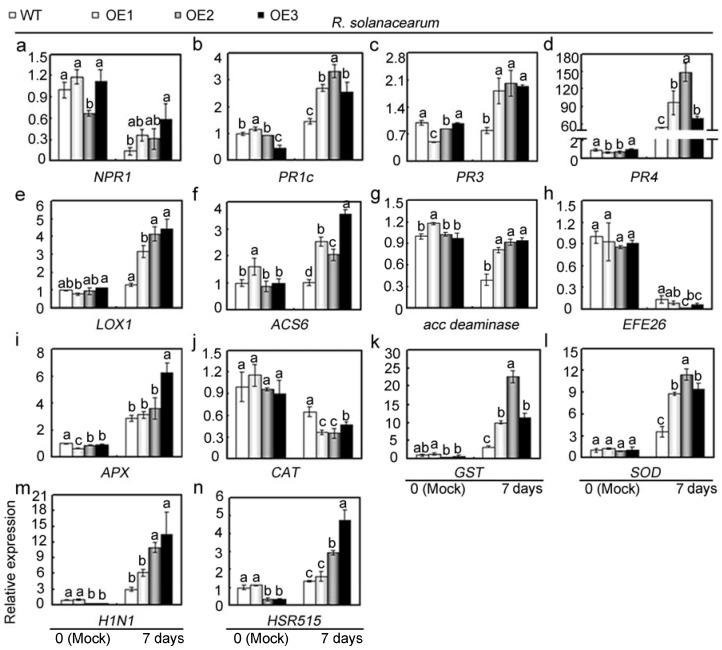
Relative transcript levels of defence-related genes in WT and *GhMAP3K65*-overexpressing plants after *R. solanacearum* infection. (**a**–**d**) Relative transcript levels of the SA-responsive genes *NPR1*, *PR1c*, *PR3,* and *PR4.* (**e**) Relative transcript levels of the JA-responsive gene *LOX1*; (**f**–**h**) Relative transcript levels of the ET biosynthesis-associated genes *ACS6*, *acc deaminase* and *EFE26*; (**i**–**l**) Relative transcript levels of the ROS detoxification-associated genes *APX*, *CAT*, *GST,* and *SOD*; (**m**,**n**) Relative transcript levels of the HR marker genes *H1N1* and *HSR515*. The experiments were repeated at least three times. Different letters above the columns indicate significant differences (*p* < 0.05) according to Duncan’s multiple range test.

To explore mechanisms underlying *GhMAP3K65*-regulated diseases and heat stress sensitivity, the expression levels of defence-related genes were examined after pathogen infection and heat stress, respectively. Detached leaves from untreated WT and transgenic *N. benthamiana* plants were used as mock controls. Following *R. solanacearum* infection for 3 or 7 days (Figures S5 and Figure 6), the transcription levels of the SA-related genes *NPR1*, *PR1c*, *PR3,* and *PR4*; the JA-responsive gene *LOX1*; the ET generation-related genes *ACS6*, *acc deaminase* and *EFE26* (ethylene forming enzyme 26); the ROS detoxification-related genes *APX*, *CAT*, *GST* (glutathione transferase ), and *SOD* (superoxide dismutase); and the HR-related genes *H1N1* and *HSR515* (hypersensitivity-related 515) were examined. Examined tobacco genes have been suggested to be up-regulated after pathogen infection [13,23,24]. A subset of these genes showed higher transcript levels in *GhMAP3K65*-overexpressing leaves than in WT leaves. At the two tested timepoints, these genes (*NPR1*, *PR1c*, *PR3*, *PR4*, *LOX1*, *ACS6*, *acc deaminase*, *APX*, *GST*, *SOD*, *H1N1*, and *HSR515*) were differentially up-regulated by *R. solanacearum* infection, while a second set of genes, including *EFE26* and *CAT*, was transcriptionally down-regulated by *R. solanacearum* at the two tested timepoints. Thus, *GhMAP3K65*-overexpression influenced the transcription (up- or down- regulation) of 13 defence-related genes examined after *R. solanacearum* infection. These results indicated that *GhMAP3K65*-overexpression activates the transcription of defence-related genes, and *GhMAP3K65* might therefore play a positive role in response to *R. solanacearum*.

**Figure 7:**
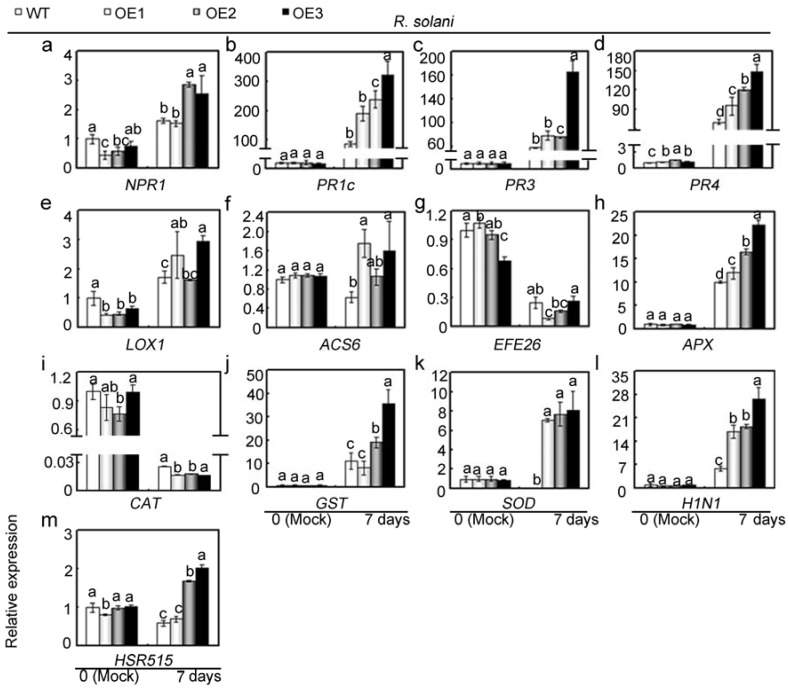
Relative transcript levels of defence-related genes in WT and *GhMAP3K65*-overexpressing plants under *R. solani* infection. (**a**–**d**) Relative transcript levels of the SA-responsive genes *NPR1*, *PR1c*, *PR3,* and *PR4*; (**e**) Relative transcript levels of the JA-responsive gene *LOX1*; (**f**,**g**) Relative transcript levels of the ET biosynthesis-associated genes *ACS6* and *EFE26*; (**h**–**k**) Relative transcript levels of the ROS detoxification-associated genes *APX*, *CAT*, *GST,* and *SOD*; (**l**–**m**) Relative transcript levels of the HR-marker genes *H1N1* and *HSR515*. The experiments were repeated at least three times. Different letters above the columns indicate significant differences (*p* < 0.05) according to Duncan’s multiple range test.

To further elucidate the role of *GhMAP3K65* in response to *R. solani* and the possible underlying mechanisms, we investigated the influence of *GhMAP3K65*-overexpression on the transcript levels of 13 defence-related genes after 3 or 7 days of *R. solani* infection (Figures S6 and Figure 7). Under *R. solani* infection, *GhMAP3K65*-overexpression differentially influenced the transcription of examined genes. A subset of these genes showed higher transcript levels in *GhMAP3K65*-overexpressing leaves than in WT leaves. Furthermore, at a minimum of one of the two tested timepoints, these genes (*NPR1*, *PR1c*, *PR3*, *PR4*, *LOX1*, *ACS6*, *APX*, *CAT*, *GST*, *SOD*, *H1N1*, and *HSR515*) were differentially up-regulated by *R. solani* infection. A second set of genes, including *EFE26* and *CAT*, was transcriptionally down-regulated by *R. solani* infection at a minimum of one of the two tested timepoints. These results indicated that *GhMAP3K65*-overexpression activates the transcription of defence-related genes, and *GhMAP3K65* might therefore play a positive role in response to *R. solani*.

**Figure 8:**
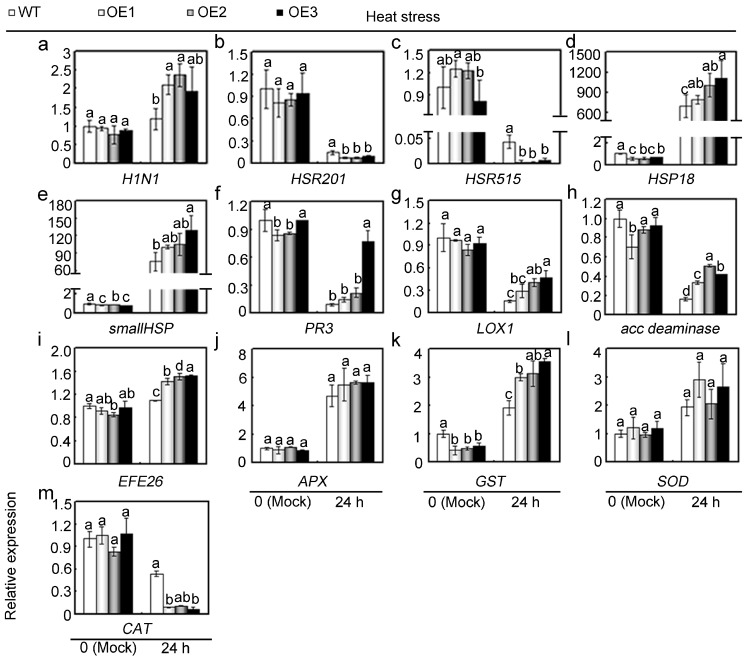
Relative transcript levels of defence-related genes in WT and *GhMAP3K65*-overexpressing plants under heat stress treatment. (**a**–**c**) Relative transcript levels of the HR-marker genes *H1N1*, *HSR201,* and *HSR515*; (**d**,**e**) Relative transcript levels of the heat-shock genes *HSP18* and *smallHSP*; (**f**) Relative transcript levels of the SA-responsive gene *PR3*; (**g**) Relative transcript levels of the JA-responsive gene *LOX1*; (**h**,**i**) Relative transcript levels of the ET biosynthesis-associated genes *acc deaminase* and *EFE26*; (**j**–**m**) Relative transcript levels of the ROS detoxification-associated genes *APX*, *GST*, *SOD,* and *CAT*. The experiments were repeated at least three times. Different letters above the columns indicate significant differences (*p* < 0.05) according to Duncan’s multiple range test.

To further ascertain possible mechanisms underlying *GhMAP3K65*-mediated reduction of thermotolerance observed in *GhMAP3K65*-overexpressing plants, the transcript levels of 13 defence-related genes were examined in WT and *GhMAP3K65*-overexpressing plants via qRT-PCR after heat stress for 24 h (Figure 8). Under heat stress, *GhMAP3K65*-overexpression differentially affected the transcript levels of defence-related genes. Most of these genes showed enhanced transcription levels in *GhMAP3K65*-overexpressing plants compared with those in WT plants after heat stress. Up-regulated genes included the HR-related gene *H1N1*; the heat shock-related genes *HSP18* and *smallHSP*; the SA-responsive gene *PR3*; the JA-responsive gene *LOX1*; the ET biosynthesis-related genes *acc deaminase* and *EFE26*; and the ROS detoxification-related genes *APX*, *GST*, and *SOD*. Downregulated transcription of *HSR201*, *HSR515,* and *CAT* was observed after heat stress, which was caused by *GhMAP3K65*-overexpression. These results indicated that *GhMAP3K65*-overexpression activates the transcription of defence-related genes, and *GhMAP3K65* might therefore play a positive role in response to heat stress.

### 2.7. Overexpression of GhMAP3K65 Impaired Ligin Biosynthesis and Stomatal Immunity in the Leaves of N. benthamiana

**Figure 9:**
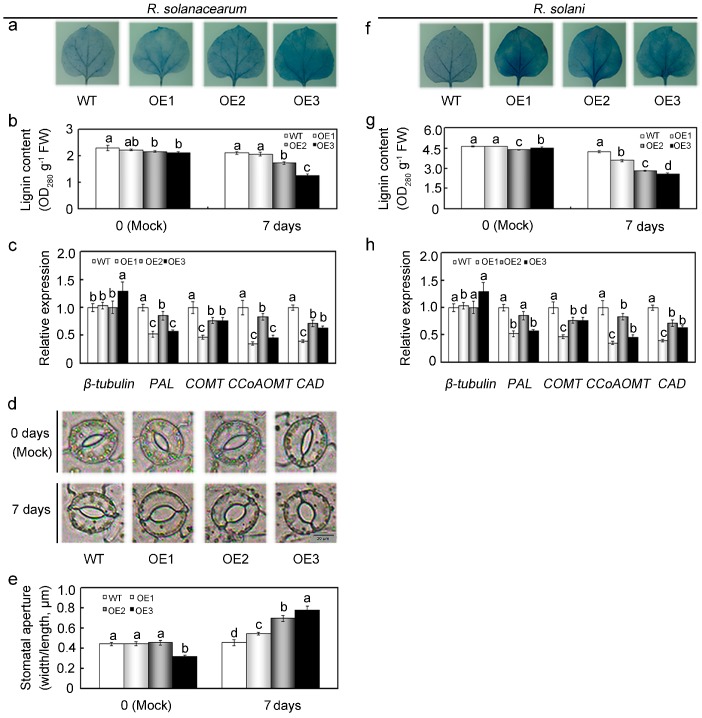
Ligin biosynthesis and stomatal immunity in the leaves of *N. benthamiana* impaired by *GhMAP3K65*-overexpression. (**a**,**f**) Leaf symptoms of WT and *GhMAP3K65*-overexpressing plants after infection with *R. solanacearum* and *R. solani* without leaf injuries; (**b**,**g**) Lignin content was analyzed from WT and *GhMAP3K65*-overexpressing lines under pathogen infection; (**c**,**h**) The expression levels of the *β-tubulin*, *PAL*, *COMT*, *CCoAOMT,* and *CAD* genes were examined in leaves via qRT-PCR; (**d**,**e**) Stomatal changes were observed using a microscope after *R. solanacearum* infection. The experiments were repeated at least three times. Different letters above the columns indicate significant differences (*p* < 0.05) according to Duncan’s multiple range test.

*GhMAP3K65*-overexpression enhanced susceptibility to pathogen infection and heat stress in transgenic *N. benthamiana* plants. Nevertheless, *GhMAP3K65* activated the transcription of defence-associated genes, which demonstrated that *GhMAP3K65* might play a positive role in the responses to pathogen infection and heat stress. Thus, we concluded that *GhMAP3K65* might also participate in other biological processes, and our expression profiling results provided some clues in this regard. *GhMAP3K65* can be induced significantly and continuously by GA_3_ (Figure 2g), and expression profiles of a gene often imply its biological function [25]. Phytohormones act as endogenous messengers and organize various signalling transduction pathways, thus allowing plants to respond to stresses [26,27,28], whereas GA plays a significant role in regulating growth and developmental processes in plants [29]. Therefore, we concluded that *GhMAP3K65* plays a significant role in modulating plant growth and development. Uninjured leaves were exposed to suspensions of pathogen for 7 days, and cell death in infected leaves was observed via Trypan blue staining. Leaves of transgenic *N. benthamiana* plants were more deeply stained than those of WT plants after pathogen infection (Figure 9a,f), revealing that *GhMAP3K65*-overexpressing plants were more significantly infected by pathogen and less competent to withstand pathogen attack. Cell wall is an obstacle that protects cells from pathogen infection. To investigate the potential effects of *GhMAP3K65* may have on cell wall traits, lignin content was analyzed from WT and *GhMAP3K65*-overexpressing plants. No obvious changes were observed in the lignin content of WT and *GhMAP3K65*-overexpressing lines under normal conditions, whereas the lignin content of *GhMAP3K65*-overexpressing lines was significantly decreased relative to that of WT plants after pathogen infection (Figure 9b,g). The transcript levels of *β-tubulin*, encoding tubulin, were not significantly different between WT and *GhMAP3K65*-overexpressing lines (Figure 9c,h). Lignin can strengthen defensive ability of cell wall, and several key enzymes involved in lignin biosynthesis, including *PAL* (phenylalanine ammonia-lyase), *COMT* (caffeic acid 3-O-methyltransferase-like), *CCoAOMT* (caffeoyl-CoA O-methyltransferase 2), and *CAD* (cinnamyl alcohol dehydrogenase 1), were therefore examined. The transcript levels of *PAL*, *COMT*, *CCoAOMT,* and *CAD* were lower in *GhMAP3K65*-overexpressing plants than in WT plants, indicating that *GhMAP3K65*-overexpression influences lignin biosynthesis (Figure 9c,h). Stomata have been shown to play an important role in plant defense as a part of the innate immune response [30]. Degree of stomatal opening in *GhMAP3K65*-overexpressing plants was not significantly different from that in WT plants under normal conditions. Nevertheless, extent of stomatal opening observed in *GhMAP3K65*-overexpressing plants was greater than that observed in WT plants after *R. solanacearum* infection (Figure 9d,e). These results indicated that *GhMAP3K65*-overexpression impaired ligin biosynthesis and stomatal immunity in the leaves of *N. benthamiana*.

### 2.8. Overexpression of GhMAP3K65 Repressed Growth and Development of the Root System

**Figure 10:**
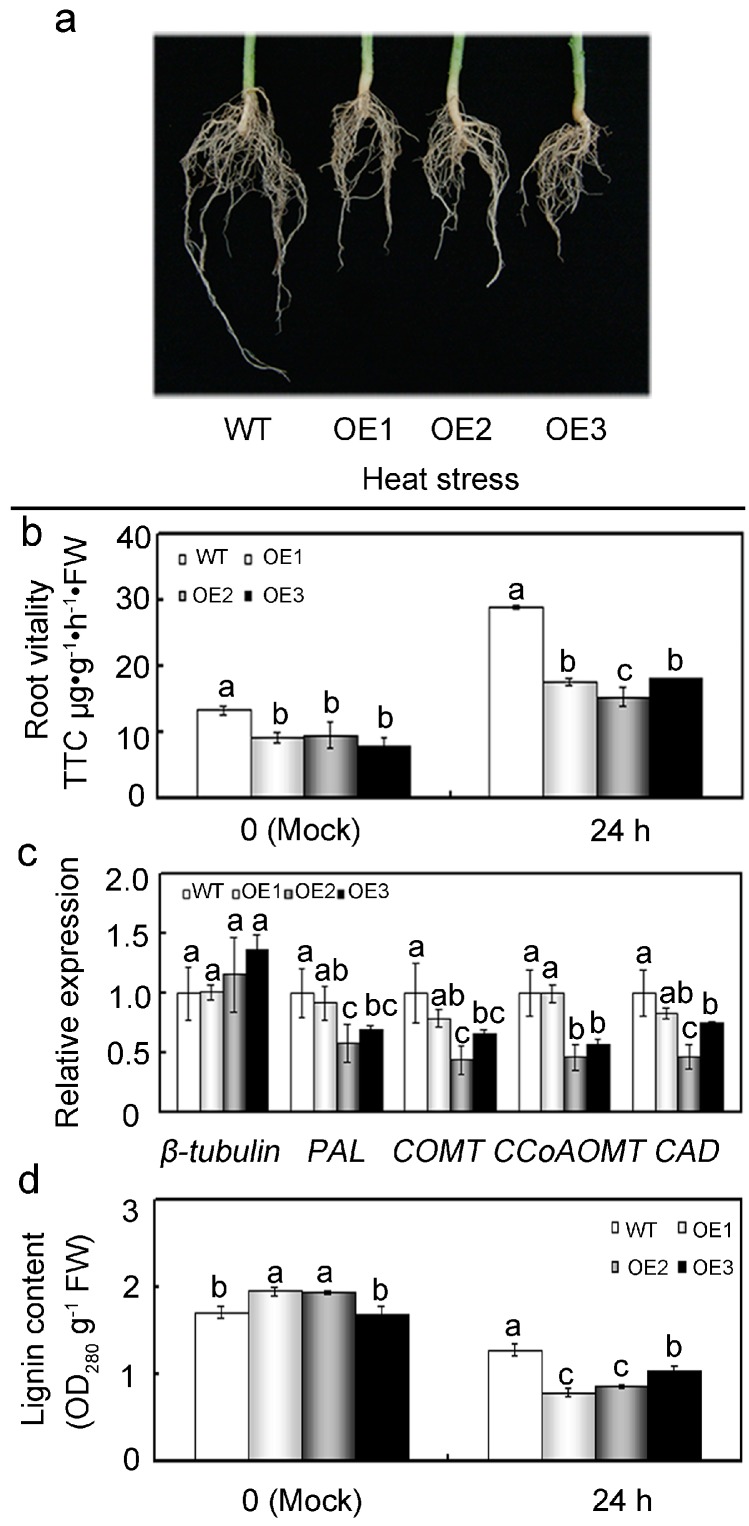
Defects in roots caused by *GhMAP3K65*-overexpression. (**a**) Root system was photographed after being grown in soil for 8 weeks; (**b**) Root vitality of WT and *GhMAP3K65*-overexpressing lines; (**c**) The expression levels of *β-tubulin*, *PAL*, *COMT*, *CCoAOMT,* and *CAD* genes were examined in leaves via qRT-PCR; (**d**) Lignin content was analyzed from WT and *GhMAP3K65*-overexpressing lines under heat stress. The experiments were repeated at least three times. Different letters above the columns indicate significant differences (*p* < 0.05) according to Duncan’s multiple range test.

Many Raf-like MAP3Ks participate in growth and development of plant root system. Therefore, root phenotypes of WT and *GhMAP3K65*-overexpressing plants were monitored (Figure 10a). Root vitality of a plant reflects growth and development of its root system, providing a comprehensive index of root vitality. Root vitality directly affects absorption and utilization of mineral nutrients and water, which play decisive roles in growth and development of entire plant. Root vitality of *GhMAP3K65*-overexpressing lines was significantly decreased compared with that of WT plants after heat stress relative to mock control (Figure 10b). Several lignin biosynthesis genes were detected in roots, and the transcript levels of *PAL*, *COMT*, *CCoCOMT,* and *CAD* were found to be lower in *GhMAP3K65*-overexpressing lines than in WT plants after heat stress (Figure 10c). To investigate the potential effects of *GhMAP3K65* may have on cell wall traits, lignin content was analyzed from WT and *GhMAP3K65*-overexpressing plants. No evident changes were observed in the lignin content of WT and *GhMAP3K65*-overexpressing lines under normal conditions, whereas lignin content of *GhMAP3K65*-overexpressing lines was significantly decreased relative to that of WT plants after heat stress (Figure 10d). These results demonstrated that *GhMAP3K65*-overexpression inhibits growth and development of the root system.

## 3. Discussion

Many studies have indicated that members of MAPK family participate in defensive reactions to environmental stresses. Expression of numerous MAPK genes is influenced by pathogen infection [31], high salinity [32], cold [33], drought, and heat stress [34]. While numerous MAP3Ks have been identified in various plant genomes, biological functions of only a few have been studied. In this study, multiple sequence alignment and evolutionary tree analyses confirmed that *GhMAP3K65* is a Raf-like MAP3K gene. In addition, subcellular localization analysis of GhMAP3K65 suggested that the fusion protein localized in the cytoplasm and nucleus, indicating that GhMAP3K65 may function in both areas. Expression profiles of a gene often imply its biological function [25]. *GhMAP3K65* transcription was induced by pathogen infection, heat and diverse signalling molecules in cotton, indicating that *GhMAP3K65* may play a role in linking signalling pathways to plant defence responses.

Raf-like MAP3Ks play roles in response to biotic and abiotic stresses. This group of kinases includes *GhMAP3K40* (mediates reduced tolerance to biotic and abiotic stresses) [12], *DMS1* (mediates drought resistance) [35] and *Raf43* (required for tolerance to multiple abiotic stresses) [2]. To broaden a previous analysis of *GhMAP3K65*, its roles in the responses to pathogen infection and heat stress were examined through loss- and gain-of-function experiments. Silencing of *GhMAP3K65* enhanced resistance to pathogen infection and heat stress in cotton. In contrast, overexpression of *GhMAP3K65* enhanced susceptibility to pathogen infection and heat stress in *N. benthamiana*.

Transcript accumulations of HR- and PR-associated genes are representative of plant defence responses and act as markers of pathogen infection. In tobacco plants, *H1N1*, *HSR201,* and *HSR515* are generally up-regulated in HR provoked after pathogen infection [36]. Because *GhMAP3K65*-overexpression increased transcript levels of *H1N1*, *HSR515*, *PR1c*, *PR3,* and *PR4*, it was expected to play a positive role in response to pathogen infection; however, *GhMAP3K65*-overexpressing plants instead suffered more severe harm. Plants have been reported to respond to pathogen infection with a burst of ROS. Under biotic stress, ROS act as signalling molecules to activate pathogenesis-related proteins and systemic acquired resistance in cells adjacent to the infection site to prevent further pathogen spread [37,38]. Low levels of ROS serve as signalling molecules that play important roles in plant immunity. However, ROS can also induce high levels of cell death [39]. The accumulation of ROS during plant infections by pathogens has been implicated in susceptible responses against these pathogens [25,40], and H_2_O_2_ is a typical ROS. This study demonstrated that *GhMAP3K65*-overexpressing plants accumulated more H_2_O_2_ than WT plants after pathogen infection. However, the transcript levels of ROI-detoxifying enzymes, such as *APX*, *GST,* and *SOD*, were either constitutively higher in overexpressing plants or induced after pathogen infection, indicating that *GhMAP3K65* activated the antioxidant system. The results were similar to those achieved using defense-related gene assays—the overexpressing plants were expected to be more resistant to pathogen infection, but they instead suffered more severe damage.

*GhMAP3K65*-overexpression increased susceptibility of *N. benthamiana* plants to heat stress. A specific relationship between accumulation of heat shock proteins (HSPs), particularly small HSPs, and thermotolerance was demonstrated previously [41]. We observed that *GhMAP3K65*-overexpression enhanced the transcription of *HSP18* and *smallHSP* after heat stress compared with that in WT plants. In addition, *GhMAP3K65*-overexpression was observed to increase the transcript levels of *APX*, *GST,* and *SOD* significantly. Induction of ROS, such as APX, GST, and SOD, reportedly plays a role in thermotolerance [42,43,44]. *GhMAP3K65*-overexpression enhanced the transcription of heat resistance-related genes, such as *HSP18* and *smallHSP*, and ROI-detoxifying enzymes such as *APX*, *GST,* and *SOD*, which should have demonstrated the positive role of *GhMAP3K65* in response to heat stress. However, *GhMAP3K65*-overexpressing plants suffered more serious damage than WT plants after heat stress.

*GhMAP3K65*-overexpression enhanced *N. benthamiana* susceptibility to pathogen infection and reduced its tolerance to heat stress, which was not consistent with increased the transcript levels of defence-related and *HSP* genes. Similarly, many studies have shown that a single MAP3K may play a role in response to diverse environmental stresses and function in several seemingly different signalling pathways. For instance, *GhMAP3K40* functions in response to both biotic and abiotic stresses (e.g., drought, *R. solanacearum* and *R. solani*), playing a negative role in response to drought, *R. solanacearum* and *R. solani* [12]. Additionally, *Raf43* is involved in the regulation of innate immunity in *Arabidopsis* [2]. Therefore, *GhMAP3K65* may play a significant role in response to pathogen infection and heat stress.

While research regarding disparate triggering phenomena and their molecular bases has received increasing interest [45,46]. However, the underlying molecular mechanisms triggered by plant stress remain widely unknown [47]. *GhMAP3K65* may play a role in crosstalk between pathogen infection and heat stress signalling pathways mediated by SA, JA, and ET. It is often observed that pathogen infection tends to be more severe under the environmental challenges of higher temperature and humidity. We observed that the transcription of *HSR201*, *HSR515,* and *CAT* is inhibited in the response to heat stress compared with that in WT plants, demonstrating a negative influence of heat stress on plant defence responses. However, *GhMAP3K65*-overexpression increased the transcript levels of the SA-responsive gene *PR3*; the JA-responsive gene *LOX1*; the ET production-associated genes *acc deaminase;* and *EFE26*; and the ROS detoxification-associated genes *APX*, *GST,* and *SOD* in response to heat stress compared with their levels in WT plants. Therefore, *GhMAP3K65*-overexpression appears to reverse the negative influences of high temperature and humidity in the response to pathogen infection.

Previous studies have demonstrated abundant crosstalk between pathogen infection and heat stress signalling pathways [28,48]. R-genes, such as *cf-4* and *Cf-9* in tomato have been reported to be involved in such crosstalk [49]. In this study, the transcript levels of *HSP18* and *smallHSP* were significantly elevated by *GhMAP3K65*-overexpression in the response to heat stress, providing strong evidence that heat-induced HSPs play roles in crosstalk between pathogen infection and heat stress mediated by *GhMAP3K65*.

The expression levels of hormones SA, JA, and ET are constantly elevated in the response to pathogen infection. The balance of these hormones relies on discriminating the pathogen and plays a crucial role in the fine mediation of appropriate plant defence responses [1]. SA mediates fundamental defence responses to biotrophic pathogen infection, while JA generally controls significant defence responses to necrotrophs [50]. The hormones SA, JA, and ET have been demonstrated to induce different *PR* genes in plants and work either antagonistically or synergistically in defence signalling pathways depending on their concentration [51,52]. In addition to their participation in disease defence signalling pathways, SA, JA, and ET have been implicated in thermotolerance [53,54]. We demonstrated that *GhMAP3K65* transcription was induced by exogenous application of the plant hormones SA (2 mM), JA (100 μM), and ET (5 mM) and that *GhMAP3K65*-overexpression increased the transcript levels of the SA-associated gene *PR3*, the JA-responsive gene *LOX1*, and the ET production-related genes *acc deaminase* and *EFE26* in response to heat stress. Hence, we conclude that SA, JA, and ET modulate the expression of *GhMAP3K65*, influencing the expression of downstream defence- and thermotolerance-associated genes.

*GhMAP3K65*-overexpressing plants showed enhanced susceptibility to pathogen infection and heat stress. Nevertheless, *GhMAP3K65* was demonstrated to play a positive role in plant defence responses. Additionally, *GhMAP3K65* transcription was induced by gibberellin. These results promoted evaluation of the putative role of *GhMAP3K65* in modulating plant growth and development. After pathogen infection, uninjured leaves of *GhMAP3K65*-overexpressing plants exhibited more severe cell death than those of WT plants. These results indicated that *GhMAP3K65*-overexpressing plants are less competent to withstand pathogen attack, which may result from impaired ligin biosynthesis and stomatal immunity in the leaves of *N. benthamiana*. Lignin confers rigidity to cell walls and is therefore associated with tolerance to biotic and abiotic stresses and the mechanical stability of plants [55]. The cell wall constitutes the first line of plants defence against pathogens, such as bacteria and fungi [54,56]. Lignin solidifies the cell wall, providing a nondegradable barrier to pathogens and is therefore thought to enhance protection against such biotic stresses [57]. An increase in lignification is often observed in response to pathogen attack [57]. Decreased lignin content has been exhibited to hinder plant growth and development [58,59]. However, *GhMAP3K65*-overexpression decreased lignin content in *N. benthamiana* plants compared with that in WT plants. Hence, several key enzymes involved in lignin biosynthesis were examined. However, transcription of these lignin biosynthesis genes was suppressed in *GhMAP3K65*-overexpressing plants, which indicated that *GhMAP3K65*-overexpression influences lignin biosynthesis. Stomata have traditionally been assumed to merely be passive ports for pathogen entry. However, stomata are now clearly viewed as an integral part of the plant immune system, and regulation of their aperture prevents pathogen entry via leaves [60]. Bacterial pathogens can successfully manipulate stomatal immunity to promote host infection [30]. *R. solanacearum* enhanced stomatal opening in *GhMAP3K65*-overexpressing plants compared with that in WT plants. These results showed that *GhMAP3K65*-overexpression decreased lignin content and lignin biosynthesis, and increased stomatal opening, therefore impairing ligin biosynthesis and stomatal immunity in the leaves of *N. benthamiana*, which enhanced susceptibility to pathogen infection. In addition, root phenotype and root vitality were significantly different in the WT and *GhMAP3K65*-overexpressing lines, providing an explanation for the enhanced susceptibility of transgenic *N. benthamiana* plants to heat stress. Numerous studies have indicated that roots are more sensitive to heat stress than other plant tissues, suggesting that high soil temperature is more detrimental to whole-plant growth than high air temperature [61]. The expression levels of cell wall-associated genes can be altered in response to heat stress treatment. A transcriptomic study carried out in Chinese cabbage (*Brassica rapa* L.) showed that several cell wall-associated genes could play roles in the acquisition of thermotolerance [62]. Heat stress can mediate changes in secondary cell wall metabolism. Lignin biosynthesis can be altered during the application of elevated temperature. Thus, the expression levels of some key enzymes involved in lignin biosynthesis were examined. Transcription of these lignin biosynthesis genes was inhibited in *GhMAP3K65*-overexpressing plants, which indicated that *GhMAP3K65*-overexpression influences lignin biosynthesis. Moreover, *GhMAP3K65*-overexpression decreased lignin content in *N. benthamiana* plants compared with that in WT plants. Overall, *GhMAP3K65* negatively modulates plant growth and development and therefore results in enhanced susceptibility to pathogen infection and heat stress.

In conclusion, *GhMAP3K65* plays roles in the responses to pathogen infection and heat stress mediated by SA, ET, and JA. Although complex regulatory mechanisms involving Raf-like MAP3K proteins in cotton remain unclear, this work provides further insight into the regulatory mechanisms of a Raf-like MAP3K65 protein.

## 4. Materials and Methods

### 4.1. Plant Growth and Stress Treatments

Cotton (*Gossypium hirsutum* L. cv. lumian 22) seeds were germinated in moist gauze, and seedlings were transferred to hydroponic culture for growth in a light chamber at 26 ± 1 °C, with a 16 h light/8 h dark cycle and relative humidity of 60–75%. Seven-day-old cotton seedlings were subjected to diverse treatments described below. For pathogen infection, seedlings were inoculated with suspensions of bacterial pathogen *R. solanacearum* (OD_600_ = 0.6–0.8) and conidial suspensions of fungal pathogen *R. solani* (10^5^ conidia/mL) using root-dipping method. For heat stress, seedlings were exposed to heat stress in a 42 °C incubator for 24 h at 100% humidity and darkness. For signalling molecules, cotton leaves were sprayed with 2 mM SA, 100 µM MeJA, 5 mM ET and 500 µM GA_3_. Regarding the sample collection time points, 8 o’clock a.m. correspond to 0 h, 9 o’clock a.m. correspond to 1 h, etc. Samples collected at appropriate times were snap-frozen in liquid nitrogen and then stored at −80 °C for subsequent analyses. *N. benthamiana* seedlings at three- or four-leaf stages were transplanted into pots with wet soil and maintained under glasshouse conditions at 25 °C with a 16 h light/8 h dark photoperiod. Uniformly grown seedlings were used in subsequent studies.

### 4.2. Isolation of GhMAP3K65 Gene, Vector Construction, and Genetic Transformation

*GhMAP3K65* was obtained as described previously [63]. Vector construction and genetic transformation were carried out according to previously described methods [32]. T_2_ progenies of WT and *GhMAP3K65*-overexpressing plants were subjected to subsequent functional analyses.

### 4.3. Subcellular Localization of GhMAP3K65

*GhMAP3K65* ORF without stop codon was fused to 5′-terminal end of GFP to construct pROKII-GhMAP3K65-GFP, which was regulated by CaMV35S promoter. Positive control pROKII-GFP construct and pROKII-GhMAP3K65-GFP construct were transformed into *Agrobacterium tumefaciens* strain GV3101, respectively. *Agrobacterium* cells collected via centrifugation were resuspended in infiltration buffer (for 100 mL: 1 mL of 1 M MES, pH 5.6; 200 μL of 0.1 M acetosyringone (AS); and 100 μL of 1 M MgCl_2_) and adjusted to a final OD_600_ of 1.0. After cultivation for 3 h, *Agrobacterium* mixture was inoculated into *N. benthamiana* leaves with 1 mL sterile syringe. Lower epidermis cells were observed using two-photon laser confocal microscope.

### 4.4. RNA Extraction and Quantitative PCR

Total RNA was obtained from samples using improved cetyltrimethylammonium bromide (CTAB) method [64] and TRIzol reagent (TaKaRa, Dalian, China) and then used for first-strand cDNA synthesis with EasyScript First-strand cDNA Synthetic SuperMix (TransGen Biotech, Beijing, China). To examine *GhMAP3K65* transcript levels under different treatments, qRT-PCR was carried out with SYBR PrimeScript^TM^ RT-PCR Kit (TaKaRa) in 20 µL reaction volume, using CFX96TM Real-time System (Bio-Rad, Hercules, CA, USA) following experimental procedures described previously by Yu et al. [65]. *G. hirsutum ubiquitin* (*UBI*) and *N. benthamiana β-actin* were employed as control genes. The primer pairs used for qRT-PCR are listed in Table S1.

### 4.5. Virus-Induced Gene Silencing (VIGS) of GhMAP3K65 in Cotton

To silence *GhMAP3K65* in cotton, Tobacco Rattle Virus (TRV)-based VIGS system was employed. A fragment of *GhMAP3K65* transcribed region was inserted into multiple cloning site of pTRV-RNA2 plasmid to generate pTRV2-*GhMAP3K65* constructs. The pTRV1, pTRV2, and pTRV2-*GhMAP3K65* constructs were then transformed into *Agrobacterium tumefaciens* strain GV3101, respectively. *Agrobacterium* carrying pTRV2 or pTRV2-*GhMAP3K65* construct was mixed with pTRV1 strain (1:1 ratio, OD_600_ = 1.0) and co-infiltrated into cotton cotyledons using 1 mL sterile syringe [20]. VIGS efficiency was assessed via qRT-PCR, and silenced cotton plants were transplanted into soil mixture for subsequent functional analyses. CK and VIGS cotton plants were inoculated with pathogen infection for 7 days using root-dipping method. CK and VIGS cotton plants were exposed to heat stress in a 42 °C incubator for 24 h at 100% humidity and darkness.

### 4.6. Pathogen Infection

*R. solanacearum* was incubated overnight at 37 °C in Luria–Bertani (LB) broth in shaker, then collected via centrifugation and resuspended in sterile tap water. *R. solani* was cultured on potato dextrose agar (PDA) medium in incubator at 28 °C for 2 weeks, after which its spores were suspended in 1% glucose. For pathogen infection, detached leaves of 2-month-old WT and *GhMAP3K65*-overexpressing plants were inoculated with suspensions of *R. solanacearum* (OD_600_ = 0.6–0.8) and conidial suspensions of *R. solani* (10^5^ conidia/mL) using needleless syringe. Detached leaves from untreated WT and transgenic *N. benthamiana* plants were used as mock controls. Inoculated leaves were maintained in a glass culture dish and lesions could be observed 7 days after inoculation. Chlorophyll content was determined by described previously [66]. The colony forming units (cfu) of *R. solanacearum* were determined by described previously [67]. The average number of spores per cotyledon of *R. solani* was determined by described previously [68].

### 4.7. Heat Stress Treatment

To evaluate impact of *GhMAP3K65* on thermotolerance and to guarantee that necrosis resulting from heat stress was due to increased temperature and not photo-oxidative stress, WT and *GhMAP3K65*-overexpressing *N. benthamiana* plants were exposed to heat stress in a 42 °C incubator for 24 h at 100% humidity and darkness [20].

### 4.8. Histochemical Staining

For DAB staining, *N. benthamiana* leaves were incubated in DAB solution (1 mg/mL, pH 3.8) for 12 h at 25 °C in the dark. After DAB staining, *N. benthamiana* leaves were boiled for 10 min to remove chlorophyll and then soaked in 95% ethanol. The Trypan blue staining solution comprised 40 mL of absolute ethanol, 0.01 g of Trypan blue, 5 mL of lactic acid, 5 mL of glycerol, 5 g of phenol and 5 mL of double-distilled water. *N. benthamiana* leaves were soaked in Trypan blue solution, boiled for 5 min, and then incubated in Trypan blue staining solution for 12 h. In addition, *N. benthamiana* leaves were soaked in 95% ethanol to remove chlorophyll.

### 4.9. Structural Defects in Leaves

To explore whether there are structural defects in leaves, uninjured leaves from WT and transgenic *N. benthamiana* plants were selected and solutions of pathogen were smeared on both sides of the leaves. One week later, the leaves were cut and cell death was detected by Trypan blue staining.

### 4.10. Lignin Content

Leaves (0.1 g fresh weight (FW)) were triturated using liquid nitrogen, added to a 14-mL centrifuge tube and soaked in 12 mL of 95% ethanol to remove chlorophyll. The supernatant was discarded after centrifugation at 5000 rcf for 10 min. A solution comprising *N*-hexane:ethanol (2:1, 12 mL) was added to the precipitant, which was then centrifuged at 5000 rcf for 10 min. The precipitant was dried, treated with 2.5 mL of 25% acetyl bromide, incubated at 70 °C for 30 min, and cooled quickly in cold water. Next, 0.9 mL of 2 mol/L NaOH was added to the above reaction solution to stop the reaction, and 0.1 mL of 7.5 mol/L hydrochloric acid hydroxylamine and 4 mL of glacial acetic acid were added before centrifugation at 5000 rcf for 5 min. Next, 3.9 mL of glacial acetic acid was added to 0.1 mL of the supernatant, and the absorbance at 280 nm per gram of fresh sample represented the lignin content. For determining the lignin content of the root system, the same steps used for leaves were taken, except the chlorophyll was removed.

### 4.11. Determination of Root Vitality by Triphenyl Tetrazolium Chloride (TTC) Method

Root vitality was determined using a root TTC reduction method in tissues following treatment with a red-coloured insoluble complex [69,70]. A TTC solution (0.25 mL of 0.4%) and a small Na_2_S crystal were added to a 10-mL volumetric flask, which was shaken immediately after the addition of red triphenylformazan (TF) and 95% ethanol. Then, TF (0.25, 0.5, 1.00, 2.00 and 4.00 mL) and 95% ethanol were added to the 10-mL volumetric flask. A blank tube (0 mL of TF) served as the control, and a standard curve was formed. TTC reduction was examined using an improved method described by Zhang et al. [70]. Root segments (0.5 g fresh weight (FW)) were incubated for 6 h in the dark at 37 °C with a mixture comprising 10 mL of 0.4% TTC and 10 mL of tris(hydroxymethyl)aminomethane hydrochloride (Tris-HCl, pH 8.4). Next, 2 mL of 1 mol/L H_2_SO_4_ was added to the above reaction solution (except the control) to stop the reaction (control: root segments were incubated with 2 mL of 1 mol/L H_2_SO_4_, while the other operations were the same). Root segments were subsequently recovered on filter paper and washed with double-distilled water. Water-insoluble red TF was extracted from the root segments using 6 mL of 95% ethanol for 20 min. Absorbances of the extracts were measured at 485 nm to determine root vitality according to the standard curve (i.e., root vitality: TTC μg·g^−1^·h^−1^·FW).

## Supplementary Materials

Supplementary materials can be found at www.mdpi.com/1422-0067/18/11/2462/s1.
