# Supplementary material for: GhMAP3K65, a Cotton Raf-Like MAP3K Gene, Enhances Susceptibility to Pathogen Infection and Heat Stress by Negatively Modulating Growth and Development in Transgenic Nicotiana benthamiana
Source: Int J Mol Sci. 2017 Nov 21;18(11):2462. doi: 10.3390/ijms18112462 (PMC5713428; doi:10.3390/ijms18112462)
Supplement: Supplementary file 1 [file ijms-18-02462-s001.pdf]

Supplementary Figures

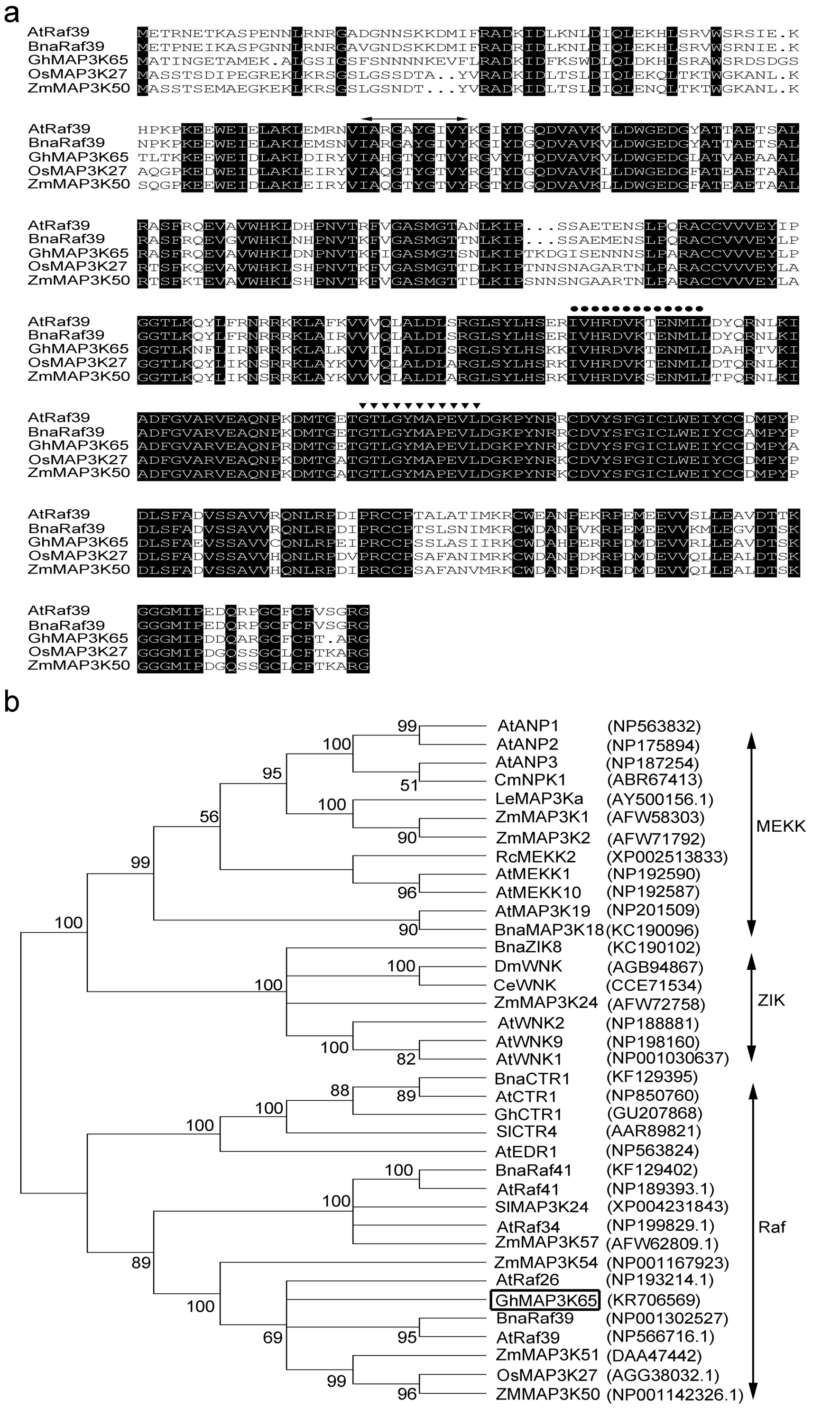

Figure S1: Sequence and phylogenetic analyses of GhMAP3K65. (a) Alignment of the GhMAP3K65 amino acid sequence with AtRaf39 (NP189393), BnaRaf39 (KF129402), OsMAP3K27 (XP_004231843) and ZmMAP3K50 (AFW62809). Identical amino acids are highlighted in black. The ATP-binding site and the Ser/Thr kinase active site are indicated with a two-headed arrow and a circle, respectively. The conserved signature motif is indicated with a triangle. (b) Phylogenetic analysis of GhMAP3K65 in relation to other

MAP3K protein. GhMAP3K65 is indicated by a frame. Numbers above or below branches represent bootstrap values (50%) from 500 replicates.

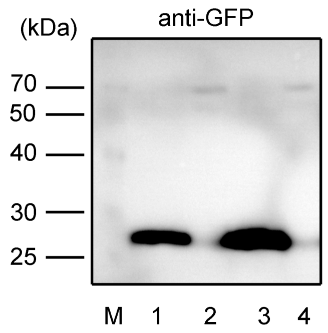

Figure S2: Western blot analyses of 35S-GFP and 35S-GhMAP3K65::GFP proteins from transiently transformed *N. benthamiana* plants using a GFP antibody. Lane 1 and 3, 35S-GFP; lanes 2 and 4, 35S-GhMAP3K65::GFP.

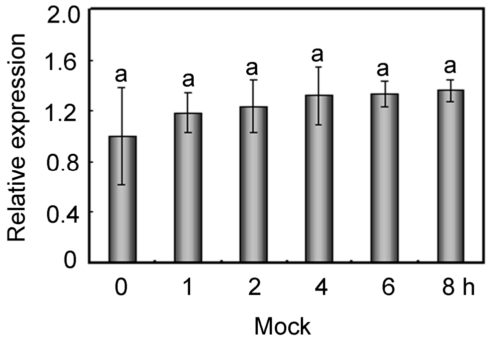

Figure S3: Expression profiles of *GhMAP3K65* in cotton under the mock control condition; seven-day-old untreated cotton seedlings were examined. Regarding the sample collection times, 8 o'clock a.m. correspond to 0 hours (h), 9 o'clock a.m. correspond to 1 h, etc. The expression profiles of *GhMAP3K65* were determined via quantitative real-time PCR (qRT-PCR). Total RNA was extracted from cotton cotyledons at the indicated time points. *GhUBI* (GenBank accession number: EU304080) was used as the internal control, and the experiments were repeated at least three times. The different letters above the columns indicate significant differences ($P < 0.05$) according to Duncan's multiple range test.

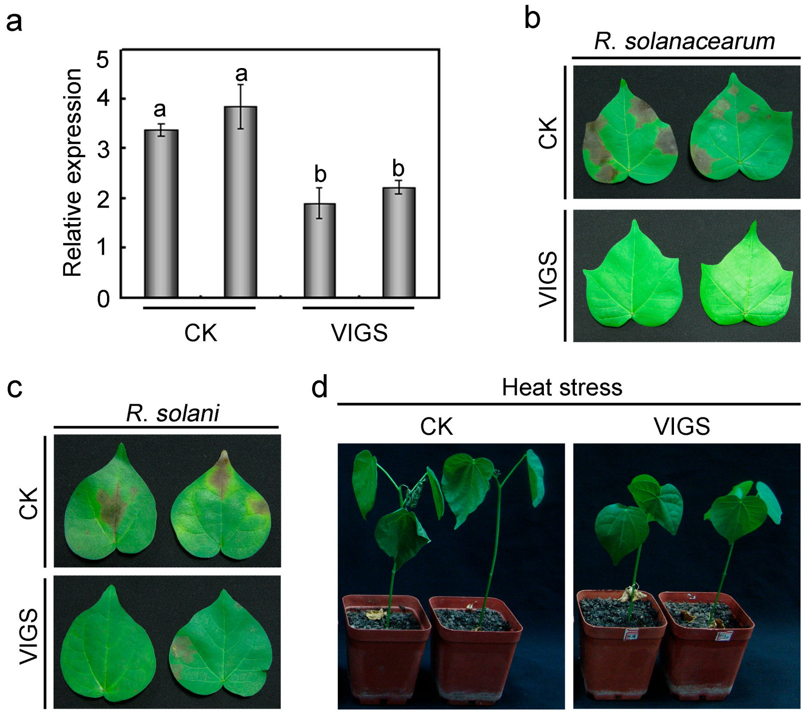

Figure S4: Loss-of-function analysis of *GhMAP3K65* in cotton. (a) Relative *GhMAP3K65* transcript levels in vector-treated (CK) and *GhMAP3K65*-silenced (VIGS) cotton plants were examined via qRT-PCR. (b-d) Representative phenotypes of CK and VIGS plants after *R. solanacearum*, *R. solani* and heat stress, respectively.

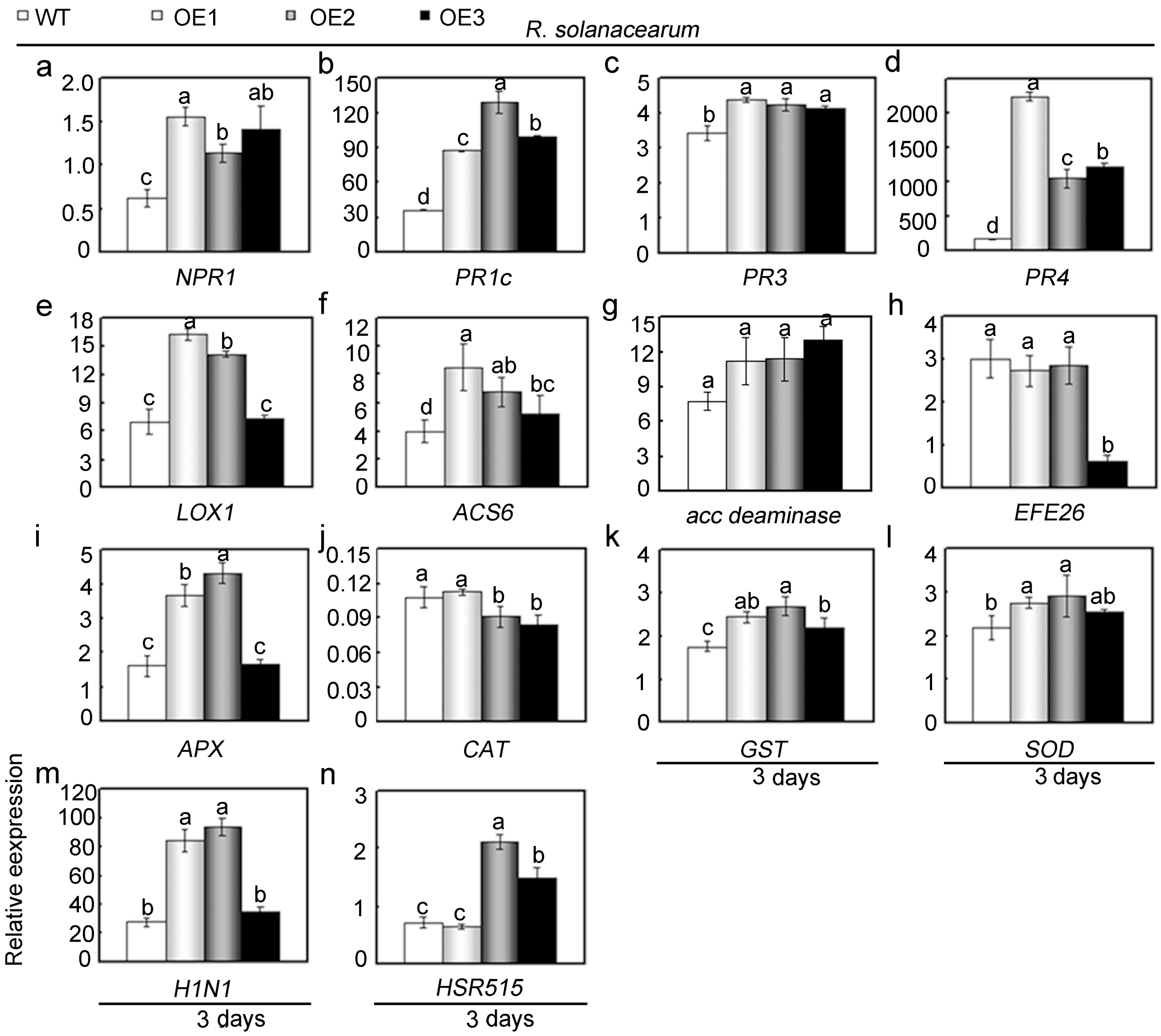

Figure S5: Relative transcript levels of defence-related genes in WT and *GhMAP3K65*-overexpressing plants under *R. solanacearum* infection 3 days. (a-d) Relative transcript levels of the salicylic acid (SA)-responsive genes *NPR1*, *PR1c*, *PR3* and *PR4.* (e) Relative transcriptional levels of the jasmonic acid (JA)-responsive gene *LOX1*. (f and g) Relative transcript levels of the ethylene biosynthesis-associated genes *ACS6* and *EFE26*. (h-k) Relative transcript levels of the reactive oxygen species (ROS) detoxification-associated genes *APX*, *CAT*, *GST* and *SOD*. (l and m) Relative transcript levels of the HR marker genes *H1N1* and *HSR515*. The experiments were repeated at least three times. Different letters above the columns indicate significant differences ($P < 0.05$) according to Duncan's multiple range test.

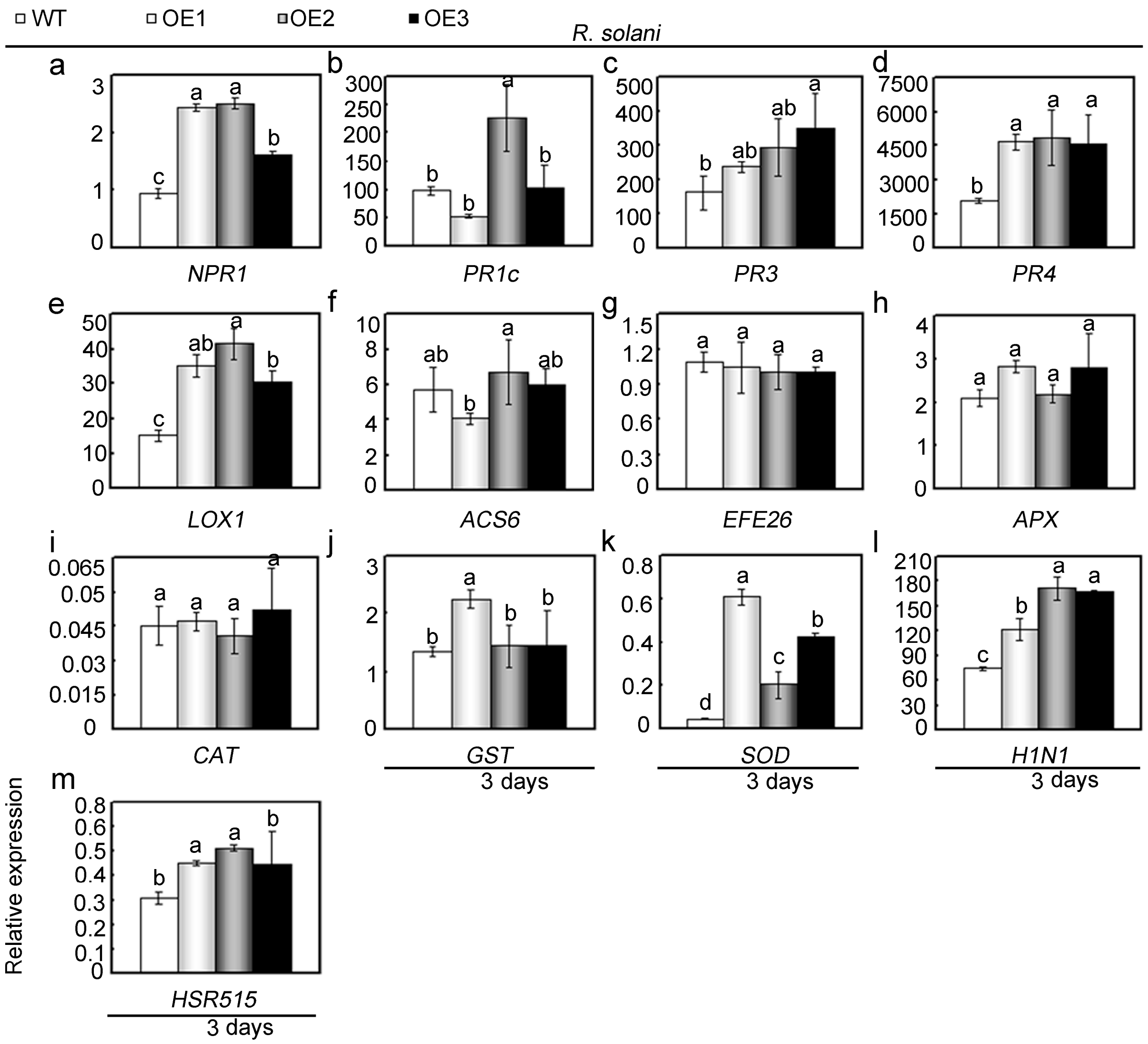

Figure S6: Relative transcript levels of defence-related genes in WT and *GhMAP3K65*-overexpressing plants under *R. solani* infection 3 days. (a-d) Relative transcript levels of the salicylic acid (SA)-responsive genes *NPR1*, *PR1c*, *PR3* and *PR4*. (e) Relative transcript levels of the jasmonic acid (JA)-responsive gene *LOX1*. (f and g) Relative transcript levels of the ethylene biosynthesis-associated genes *ACS6* and *EFE26*. (h-k) Relative transcript levels of reactive oxygen species (ROS) detoxification-associated genes *APX*, *CAT*, *GST* and *SOD*. (l-m) Relative transcript levels of the HR-marker genes *H1N1* and *HSR515*. The experiments were repeated at least three times. Different letters above the columns indicate significant differences ($P < 0.05$) according to Duncan's multiple range test.

Supplementary Tables

Primer	Primer sequence (5'→3')
Internal degenerate primers	
KP F	GTSAAGTTYATYGGBGCYTGC
KP R	TTYTTRAARGCWGCWGCATAHGC

5'-RACE premiers	
5KP F	CTCGCTTATTCCATCTTTTG
5KP R	CTTTGTAACATTCGGGTTGTC
3'-RACE premiers	
3KP F	CACCGTGATGTTAAGACCG
3KP R	CAGAACCCAAGAGACATGACC
The full-length cDNA primers	
KR F	ATGGCGACAATCAATGGTG
KR R	TCACACACATACACACTCTCTC
For the cloning of the genomic sequence	
NK1 F	ATGGCGACAATCAATGGTG
NK1 R	CTTGACCACCCGAATGTTACAA
NK2 F	CTTGACCACCCGAATGTTACAA
NK2 R	GAGACATGACCGGGGAAACAG
NK3 F	CACCGTGATGTTAAGACCG
NK3 R	GCACTCTCGGTTACATGGCTC
Primers used in promoter isolation	
QK F	GATAAGCCAACTCAGTAC
QK R	GACAACCCGAATGTTACAAAG
Primers used in constructing expressin vector	
KPBI F	GGATCCATGGCGACAATCAATGGTG
	BamH I
KPBI R	GAGCTCCACACATACACACTCTCTC
	Sac I
GFP F	GGATCCATGGCGACAATCAATGGTG
	BamH I
GFP R	GGTACCCACACATACACACTCTCTC
	Kpn I
65-CM F	GAATTCATGGCGACAATCAATGGTG
	EcoR I
65-CM R	GGTACCCTGCAACATCTTGGGTATC
	Kpn I

Quantitative real-time PCR (qRT-PCR) primers	
Nbβ-actin F	TGGACTCTGGTGATGGTGTC
Nbβ-actin R	CCTCCAATCCAAACACTGTA
GhUBI F	CCAGAAGGAATCCACTTTGC
GhUBI R	CCAGCTCACATCAGCATACG
KQ1	GGATGCACACCCTGAACGAC
KQ2	GAAACAGAAACATCCACGAGCCTG
GhNPR1 F	TCAGTTTAGACAAGCCCGAGAA
GhNPR1 R	CGTATGACCCTCTTTCAGTAGCA
GhPR1 F	TGCTGTAAATATGTGGGTTAATGAG
GhPR1 R	GAAATTGCCTGGAGGAGAATAG
GhLOX1 F	ACATGCCGAAGCCGCTGCTT
GhLOX1 R	GGGCGTATTCGGGGCCCTTG
GhACS1 F	GATGACAATACCATGGAAGTTGC
GhACS1 R	TCCACCAATGTTGAGCTTCTC
GhAPX F	TCGTTGCCGTTGAGATTAC
GhAPX R	TGGTAGCATCAGGAAGACG
GhCAT F	TGATAAGTTGCTCCAGACTCG
GhCAT R	CTTCGTGGTGATTGTTGTGA
GhH1N1 F	GCTGATGAGACATCGGAGTTTA
GhH1N1 R	CTACCATTCCCAGTGTTCAAAG
GhHSP18	GGTCGCCTACGGATTTCTC
GhHSP18	GGCGAATGAAGCTAGAAAAGT
NtNPR1 F	GGCGAGGAGTCCGTTCTTTAA
NtNPR1 R	TCAACCAGGAATGCCACAGC
NtPR1c F	CTTGTCTCTACGCTTCTC
NtPR1c R	AACACGAACCGAGTTACG
NtPR3 F	CAGGAGGGTATTGCTTTGTTAGG
NtPR3 R	CGTGGGAAGATGGCTTGTTGTC
NtPR4 F	GGAAAACGGAAAGGTAAGAAGAGG
NtPR4 R	GGACACGAGGTAGGTATCACAACAA
NtLOX1 F	GTTGAAGGTTCTATCTGGCAGTTGG
NtLOX1 R	TGTTGCGATCACGAATGGCTCTA
NtACS6 F	GCATTGTTATGAGTGGAGGGG
NtACS6 R	CAGATTCTAAGGCTTCTTTTGTGAC
Ntacc deaminase F	TCTGAGGTTACTGATTTGGATTGG
Ntacc deaminase R	TGGACATGGTGGATAGTTGCT
NtEFE26 F	CGGACGCTGGTGGCATAAT
NtEFE26 R	CAACAAGAGCTGGTGCTGGA TA

NtAPX F	CGCTCCTCTTATGCTCCGTCTT
NtAPX R	GGTGGCTCTGTCTTGTCCTCTC
NtCAT F	CAACTTCCTGCTAATGCTCCAA
NtCAT R	TGCCTGTCTGGTGTGAATGA
NtGST F	AGCACCCTTACCTTTCCCTC
NtGST R	GCTTTCCTTCACAGCAGCAT CA
NtSOD F	CAACTCCACGGCTTCCAGAC
NtSOD R	TGGGTCCTGATTAGCAGTGGT
NtH1N1 F	CGACCTAACAAAGTCAAGTTCTACG
NtH1N1 R	CTCTATCTCCCAATAAAACCAAGC
NtHSR201 F	CAGCAGTCCTTTGGCGTTGTC
NtHSR201 R	GCTCAGTTTAGCCGCAGTTGTG
NtHSR515 F	TTGGGCAGAATAGATGGGTA
NtHSR515 R	TTTGGTGAAAGTCTTGGCTC
NtHSP18 F	AGAAACCCCAGATTCCCATA
NtHSP18 R	GGCAGCCTAAACCTTCTCAT
NtsmallHSP F	TCGCCAACACTCCAACCTCTG
NtsmallHSP R	TGCCGCTGCTCCTCTCCATA
Ntβ-tubulin F	GTACACTGGTGAAGGAATGGACGAG
Ntβ-tubulin R	GACTACTACTTCCATTGACGTTGTC
NtPAL F	GGGCAGCTATGTTAGTTAGGATCAAC
NtPAL R	GGCAAACATGGAGTAACATTGTGGT
NtCOMT F	TCAGAAGAAGAGCGTAACTGCACAT
NtCOMT R	CCCACTTGTGACAAACCATACACTC
NtCCoAOMT F	TACTGCCATGGCTCTTCCCG
NtCCoAOMT R	GTAATTGTCTTTGTCAGCGTCCAC
NtCAD F	GGTTCCTGGACATGAAGTGGTG
NtCAD R	TTGCCATCAGTGTAGACATCATTGC

Table S1: Primers used in this study. The underline represents restriction enzyme cutting site.
